# Immunogammopathy Maculopathy Secondary to Waldenström’s Macroglobulinemia Complicated With Diabetic Retinopathy: A Case Report and Literature Review

**DOI:** 10.7759/cureus.41622

**Published:** 2023-07-10

**Authors:** Nayumi Ogata, Kohei Ueda, Shuichiro Aoki

**Affiliations:** 1 Ophthalmology, Tokyo Teishin Hospital, Tokyo, JPN; 2 Ophthalmology, University of Tokyo, Tokyo, JPN

**Keywords:** hyperviscosity syndrome, fluorescein angiography, waldenström macroglobulinemia, diabetic retinopathy, immunogammopathy maculopathy

## Abstract

In Waldenström’s Macroglobulinemia (WM), increased immunoglobulin M causes various signs and symptoms. It sometimes presents with macular edema. A 65-year-old WM patient with a five-year history of diabetes mellitus was evaluated for ocular complications. Fundus examination and optical coherence tomography showed retinal changes consistent with non-proliferative diabetic retinopathy and foveal detachment with intraretinal cysts in the right eye, suggesting diabetic macular edema. However, on fluorescein angiography, there was no leakage over the area of foveal detachment, which led to the diagnosis of immunogammopathy maculopathy secondary to WM for macular edema and foveal detachment. The patient’s ocular manifestation remained unchanged through a follow-up period of 11 months without therapeutic interventions. Immunogammopathy maculopathy, a rare ocular manifestation of monoclonal gammopathy, demands differentiation from other causes of macular edema in WM patients. The present case highlights the importance of fluorescein angiography, or silent macula, in diabetic patients to distinguish immunogammopathy maculopathy from diabetic macular edema.

## Introduction

Waldenström’s Macroglobulinemia (WM) is an indolent low-grade lymphoma with the infiltration of the bone marrow by clonal lymphoplasmacytic cells that produce monoclonal immunoglobulin M (IgM). The clinical presentation of WM is diverse. Some signs and symptoms are secondary to organ infiltration by clonal cells, including anemia, lymphadenopathy, and splenomegaly, whereas others are due to specific immunological and physiochemical features of monoclonal IgM, such as peripheral neuropathy, hemolytic anemia, immune complex vasculitis, and hyperviscosity syndrome [1]. At the time of diagnosis, about 14% of WM patients present with hyperviscosity syndrome, which typically leads to venous stasis manifesting in the retina as hemorrhages, microaneurysms, venous dilation, and tortuosity [2]. Patients with WM can also present with macular edema, including serous macular detachment (SMD) due to serum immunogammopathy [3]. We herein report a case of immunogammopathy maculopathy secondary to WM complicated with diabetic retinopathy.

## Case presentation

**Figure 1:**
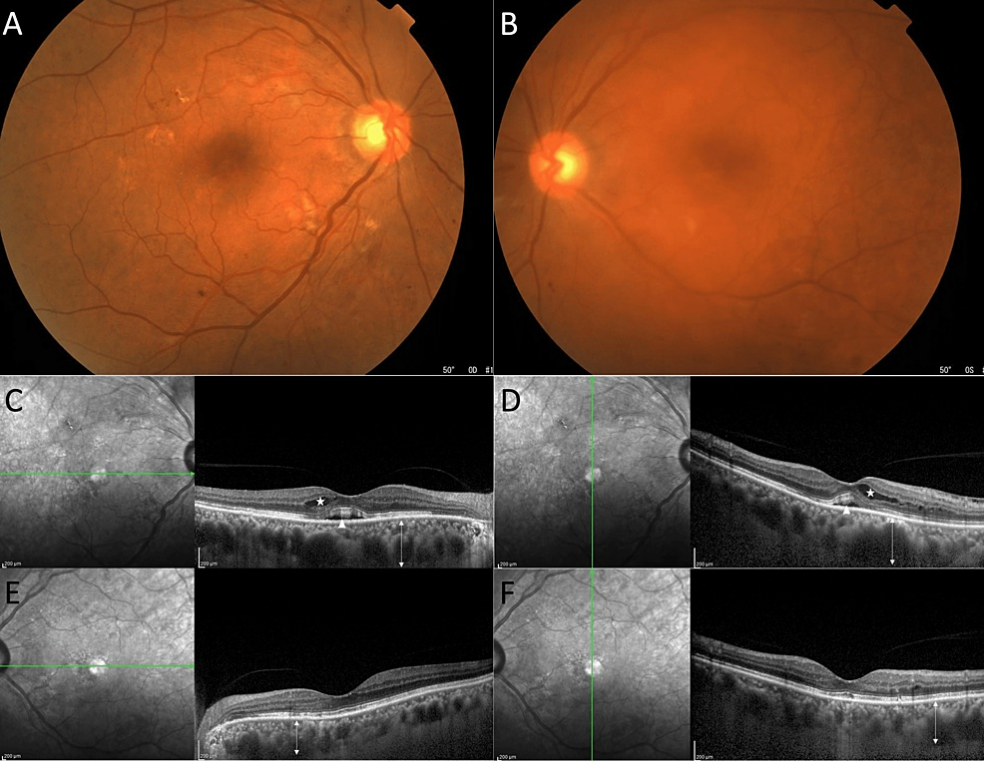
Imaging of a 65-year-old man with Waldenström’s macroglobulinemia. Fundus photographs on initial presentation of the right (A) and the left (B) eyes show dilated tortuous veins, intraretinal hemorrhages, and mild cystoid macular edema. OCT (C-F) demonstrates retinoschisis at the outer plexiform layer (★) in both eyes, foveal detachment (▲) in the right eye, and epiretinal membrane in the left eye. Choroidal thickening was seen in both eyes (↔︎). OCT – optical coherence tomography

A 65-year-old man with a five-year history of diabetes mellitus (DM) treated with oral hypoglycemic agents was referred by his family doctor to our hospital in November 2021 for evaluation of incidentally found increased IgM levels. The serum IgM level at the initial examination was 5063 mg/dL. After a detailed examination at the Department of Hematology, he was diagnosed with Waldenström’s Macroglobulinemia (WM). He was referred to our department the same month for evaluation of ocular complications of WM. He had never been examined for ocular complications of DM. His recent HbA1c level was 7.7%. Upon presentation to our department, he had no complaints about his vision. The best corrected visual acuity was 0.7 in the right eye and 0.6 in the left eye. Anterior segment examination revealed normal cornea and conjunctiva with no other remarkable findings except for a posterior subcapsular cataract in the left eye. Dilated fundus examination revealed retinal hemorrhages, cotton wool spots, and venous dilation and tortuosity in both eyes (Figure 1A, 1B). Optical coherence tomography (OCT) showed retinoschisis at the outer plexiform layer with foveal detachment in the right eye and choroidal thickening in both eyes (Figure 1C-1F).

**Figure 2:**
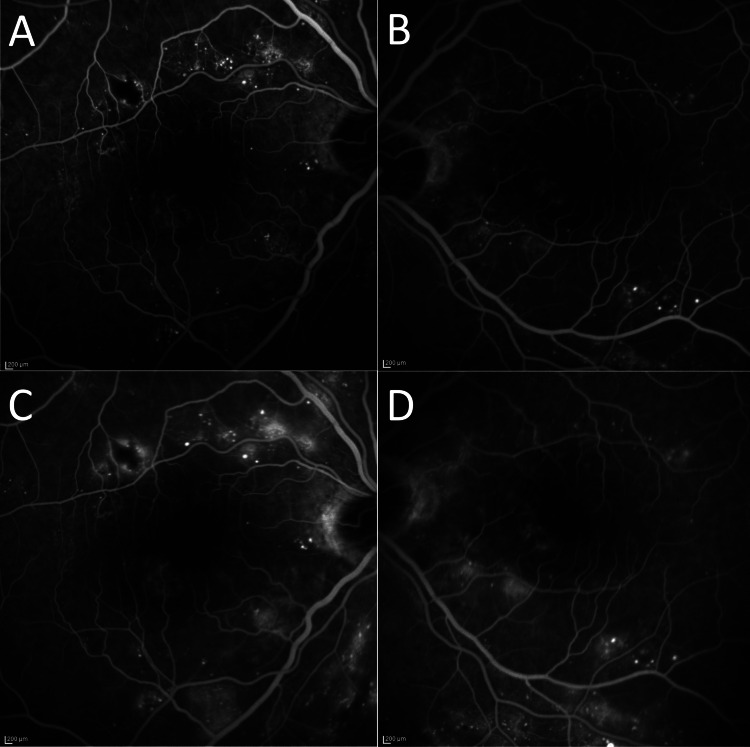
Initial Presentation of FA FA in both eyes (A, B) demonstrates mild microvascular dropouts and microaneurysms. No leakage was observed in the macula of both eyes from early-phase (A, B) to late-phase (C, D). FA – fluorescein angiography

On fluorescein angiography (FA), mild microvascular dropouts and microaneurysms were identified (Figure 2A, 2B), consistent with non-proliferative diabetic retinopathy. No leakage was observed in the macula in both eyes (Figure 2A-2D). Based on these findings, the patient was diagnosed with immunogammopathy maculopathy and non-proliferative diabetic retinopathy.

Although an attending doctor proposed possible therapeutic options for the maculopathy, such as plasmapheresis or intravitreal anti-vascular endothelial growth factor (VEGF) injections, the patient refused any treatment because he had no qualms about his vision, and no other symptoms or systemic complications. He was carefully followed up without therapeutic interventions. Visual acuity in both eyes, macular findings, and serum IgM value did not change significantly during a follow-up period from November 2021 to October 2022. The patient subsequently stopped attending appointments.

## Discussion

We encountered a case of immunogammopathy maculopathy secondary to WM complicated with diabetic retinopathy. A unique feature of this case is that it required differentiation from diabetic macular edema, a common macular complication in diabetic patients. Although venous tortuosity and retinal hemorrhages were consistent with hyperviscosity syndrome, a history of diabetes, microaneurysms, and microvascular dropouts indicated complications of diabetic retinopathy. 

Immunogammopathy maculopathy exhibits no fluorescein leakage in the macula in FA, which is described as a “silent macula.” This crucial feature distinguishes immunogammopathy maculopathy from macular edema due to the breakdown of the blood-retinal barrier, as in diabetic macular edema, age-related macular degeneration, and uveitis [4].

The pathogenesis of immunogammopathy maculopathy is not fully elucidated. A silent macula indicates that the breakdown of the blood-retinal barrier is not the sole mechanism for subretinal fluid accumulation. One mechanism of immunogammopathy maculopathy is that local ischemia related to high IgM levels may lead to outer retinal defects, providing a tract for intraretinal IgM to enter the subretinal compartment [5]. Accumulation of immunoglobulins in the neurosensory retina and subretinal space increases the osmotic pressure gradient toward the extracellular space and results in the accumulation of subretinal and intraretinal fluid [6, 7]. Indeed, several reports demonstrated the extracellular deposition of IgM in the retinal tissue and the subretinal space in patients with WM [8, 9].

Increased IgM deposition in the choroid may also contribute to choroidal thickening, as observed in the present case. Post-mortem histopathologic analysis of the eyes of a patient with light chain deposition disease detected light chain deposition in the uvea and choroid [10]. Thus, it is important to distinguish SMD in patients with WM from that resulting from central serous chorioretinopathy, which is characterized by choroidal thickening. In this regard as well, FA is a useful examination, as central serous chorioretinopathy would show fluorescein leakage due to disruption of the outer blood-retinal barrier. Hence, for patients presenting with WM and macular edema, FA helps differentiate immunogammopathy maculopathy from other more common causes of macular edema that show fluorescein leakage.

Immunogammopathy maculopathy should also be differentiated from other systemic or ocular conditions that result in macular edema without fluorescein leakage, which includes retinal dystrophy, optic nerve head pits, myopic tractional maculopathy, toxic effects of drugs such as taxanes, and systemic diseases such as Cohen syndrome [11]. The medical history and examination findings ruled out these conditions in the present case. Since some of them are treatable and others are associated with other complications, differentiation can be critical to the patient's prognosis.

Since the patient had no complaints about his vision, we carefully observed the patient without therapeutic intervention for 11 months until he stopped visits. There is no consensus on the preferred treatment for immunogammopathy maculopathy as it typically depends on the severity of the condition and the extent of vision loss [1]. Patients should be carefully followed up since previous reports of a long-term natural course are limited. Treatment of WM with systemic therapy, such as chemotherapy, immunomodulatory drugs, or targeted therapies, may help to control the underlying disease and reduce the deposition of IgM in the retina. Plasmapheresis may be used in severe cases of immunoglobulin maculopathy associated with WM to reduce the burden of abnormal immunoglobulin [12]. Local treatment options for macular edema may include intravitreal injections of steroids or anti-vascular endothelial growth factor (VEGF) agents, which are known to be effective in other forms of macular edema. However, their role in immunoglobulin maculopathy with WM remains unclear, and these have shown various results in previous reports; many cases still had persistent sub-and intraretinal fluid without improvement of visual acuity [13-15]. Reduced vascular permeability by anti-VEGF agents can prevent further accumulation of fluid, whereas the presence of IgM may result in persistent macular edema due to its osmotic effect [13]. In reported cases showing improvement in visual acuity and resolution of subretinal fluid after treatment with intravitreal bevacizumab, systemic therapy and/or plasmapheresis were concurrently applied [14, 15].

## Conclusions

Immunogammopathy maculopathy is a rare ocular manifestation of monoclonal gammopathy. It can present with macular edema with SMD and mimic other macular disorders such as diabetic macular edema. Fluorescein angiography helps differentiate these conditions.
